# Appetite Predicts Long-Term Clinical Outcomes in Patients with Acute Myocardial Infarction

**DOI:** 10.3390/jcm12196134

**Published:** 2023-09-22

**Authors:** Shun Ishibashi, Kenichi Sakakura, Tomoya Ikeda, Yousuke Taniguchi, Hiroyuki Jinnouchi, Takunori Tsukui, Yusuke Watanabe, Masashi Hatori, Kei Yamamoto, Masaru Seguchi, Hideo Fujita

**Affiliations:** Division of Cardiovascular Medicine, Saitama Medical Center, Jichi Medical University, 1-847 Amanuma, Omiya, Saitama City 330-8503, Japan

**Keywords:** percutaneous coronary intervention, acute myocardial infarction, appetite, major adverse cardiac events

## Abstract

Background: Recently, the nutritional status of patients has drawn attention in an aging society. Early studies have reported that nutritional status is related to long-term outcomes in patients with acute myocardial infarction (AMI). However, it is not necessarily simple to evaluate the nutritional status of patients with AMI. We hypothesized that appetite before discharge can be a predictor for long-term adverse cardiovascular events in patients with AMI. This retrospective study aimed to investigate whether appetite is related to long-term adverse outcomes in patients with AMI. Methods: This study included 1006 patients with AMI, and divided them into the good appetite group (*n* = 860) and the poor appetite group (*n* = 146) according to the percentage of the dietary intake on the day before discharge. Major adverse cardiac events (MACE), which were defined as a composite of all-cause death, non-fatal MI, and re-admission for heart failure, were set as the primary outcome. Results: The median follow-up duration was 996 days, and a total of 243 MACE was observed during the study period. MACE was more frequently observed in the poor appetite group than in the good appetite group (42.5% versus 21.0%, *p* < 0.001). In the multivariate COX hazard model, poor appetite was significantly associated with MACE (Hazard ratio 1.698, 95% confidence interval 1.243–2.319, *p* < 0.001) after controlling for multiple confounding factors. Conclusion: Appetite at the time of discharge was significantly associated with long-term clinical outcomes in patients with AMI. Patients with poor appetite should be carefully followed up after discharge from AMI.

## 1. Introduction

The long-term prognosis of acute myocardial infarction (AMI) has been improved by the widespread administration of primary percutaneous coronary intervention (PCI) and guideline-directed medical therapy (GDMT) [1,2]. However, some patients with AMI would have adverse events including stent thrombosis, acute heart failure, and sudden cardiac death even after the development of PCI and GDMT [3,4]. Several clinical factors are reported to be related to poor long-term prognosis in patients with AMI [5,6,7]. However, predictors for long-term outcomes of AMI are still important for stratifying patients with AMI according to the future risk after discharge.

Recently, the nutritional status of patients has drawn attention in an aging society [8,9], and several scales have been developed to assess nutritional status [10,11,12]. In fact, the nutritional status of patients with AMI may be related to long-term clinical outcomes in patients with AMI [13,14]. However, it is not necessarily simple to evaluate the nutritional status of patients with cardiovascular disease [9]. In the comparison of evaluating the nutritional status, it is simpler to assess appetite, because no blood test is required to assess appetite. There have been several reports regarding the relationship between appetite and long-term clinical outcomes in patients with heart failure or in patients after transcatheter aortic valve implantation (TAVI) [15,16]. However, there is no study that investigates the relationship between appetite and long-term outcomes in patients with AMI. We hypothesized that appetite before discharge can be a predictor of long-term clinical outcomes in patients with AMI. The purpose of this retrospective study was to investigate whether appetite is related to long-term clinical outcomes in patients with AMI.

## 2. Materials and Methods

### 2.1. Study Population

We screened consecutive patients with AMI in our hospital (Saitama Medical Center, Jichi Medical University) from January 2015 to December 2019.

### 2.2. Patient Selection

The inclusion criterion was patients with AMI who were admitted to our institution. The exclusion criteria were (1) patients who did not receive PCI for the culprit lesion of AMI, (2) patients who had in-hospital death, (3) patients without any follow-up after discharge, (4) second or more than a second AMI during the study period, when a patient experienced ≥2 AMI during the study period, (5) patients who could not take meals orally, (6) patients who underwent PCI to both culprit and non-culprit lesions of AMI simultaneously, (7) patients with unsuccessful PCI.

Appetite was assessed according to the percentage of the dietary intake on the day before discharge. Good appetite was defined as ≥70% of the dietary intake on the day before discharge, whereas poor appetite was defined as <70% of the dietary intake on the day before discharge. The final study population was divided into the good appetite group and the poor appetite group. We defined the primary endpoint as the incidence of major adverse cardiac events (MACE). MACE was defined as a composite of all-cause death, non-fatal MI, and re-admission for heart failure [10,17]. We defined the day of hospital discharge as day 1. We followed up with the study patients until meeting MACE or until January 2023 (the study end date).

### 2.3. Definition of Variables

The definition of AMI was based on the universal definition of AMI [18]. The definition of comorbidities including hypertension, dyslipidemia, and diabetes mellitus was reported elsewhere [19,20]. We collected information including body mass index, systolic blood pressure, diastolic blood pressure, heart rate at admission, hemodialysis, history of PCI, history of myocardial infarction (MI), history of coronary artery bypass graft surgery, smoking status, and medication before admission. Left ventricular ejection fraction (LVEF) was examined by echocardiography through either modified Simpson method, Teichholz method, or eyeball estimation [21]. We used the laboratory data at admission. Because we could not evaluate some laboratory data in off-hours, we substituted the earliest LDL-cholesterol or HbA1c level since admission for the data at admission. The estimated glomerular filtration rate (eGFR) was calculated by the established formula [22]. The modified KATZ index, which was defined as the sum of the six variables including feeding, continence, transferring, going to the toilet, dressing, and facial washing, was used to assess activities of daily living (ADL) prior to discharge [23]. The modified KATZ index is a 7-point scale ranging from 0 to 6, with 0 points indicating no dependence and 6 points indicating dependence on all variables [23]. We measured quantitative coronary angiography (QCA) parameters using QAngio XA 7.3 (MEDIS, Leiden, Netherlands). These parameters were calculated after the achievement of reperfusion if the lesion was totally occluded [19]. We also recorded the initial and final TIMI flow grades from coronary angiography.

### 2.4. PCI Procedures

PCI procedures were performed by using a biplane fluoroscopy system. Because our hospital is a university hospital dedicated to teaching, most PCIs for the culprit lesion of AMI are undergone by senior residents under the supervision of staff interventional cardiologists. The choice of PCI devices including guidewire, balloon, and stent was left at the discretion of staff interventional cardiologists in our catheter room. We underwent primary PCI for patients with ST-segment elevation myocardial infarction (STEMI), whereas we performed PCI to the culprit lesion in as emergent, urgent, or elective fashion for patients with non-ST-segment elevation myocardial infarction (NSTEMI). The access of primary PCI was radial, femoral, or rarely brachial arteries. First, a conventional guidewire was advanced across the culprit lesion. Then, we used a small balloon, typically a 2.0 mm balloon, or a thrombus aspiration catheter. In primary PCI, patients received 100 units/kg of unfractionated heparin. We maintained an activated coagulation time of >250 s during the procedure. For the treatment of bifurcation lesions, we dominantly performed single-stent implantation followed by kissing balloon techniques. Two-stent strategy was rarely performed to avoid late stent thrombosis. Proximal optimization techniques were preferred in most cases. Intravascular imaging devices including IVUS are utilized routinely to optimize stenting. The choice of mechanical support devices including intra-aortic balloon pumping support and veno-arterial extracorporeal membrane oxygenation was at the discretion of staff interventional cardiologists in emergent situations. All patients with AMI were treated by our coronary care unit (CCU) team, which discussed the treatment strategy of each patient at the daily CCU conference.

### 2.5. Statistical Analysis

We expressed data as mean ± standard deviation or percentage. We presented categorical variables as numbers (percentages) and compared them using the Chi-square test. For continuous variables, we performed the Shapiro-Wilk test to confirm whether the distribution of continuous variables was normal or not. We compared continuous variables with normal distribution using a student *t*-test. Otherwise, we compared continuous variables using a Mann-Whitney U test. We constructed event-free survival curves using the Kaplan−Meier method. The log-lank test was used to assess statistical differences between curves. We also carried out a multivariate Cox hazard analysis to examine the association between poor appetite and MACE after controlling for confounding factors. Variables that were significantly different (*p* < 0.05) between the good appetite group and the poor appetite group were considered confounding factors. We did not include variables with ≥5 missing values in the model. Similar variables were not included in the model simultaneously to avoid multicollinearity. We calculated hazard ratios and the 95% confidence intervals (CI). We considered *p* value < 0.05 statistically significant. We performed all analyses using statistical software, SPSS v. 25/Windows (SPSS, Chicago, IL, USA).

## 3. Results

**Figure 1:**
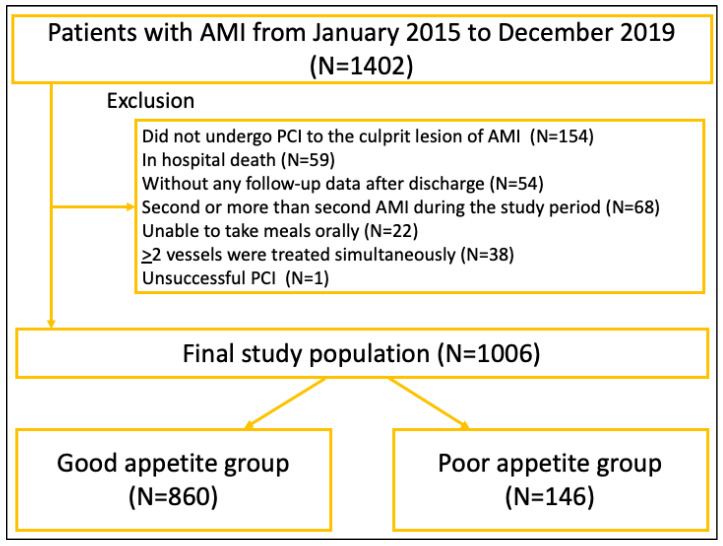
The study flow chart. Abbreviations: AMI = acute myocardial infarction.

From January 2015 to December 2019, we had 1402 patients with AMI in our medical center. We excluded 396 patients according to the exclusion criteria as follows: (1) did not undergo PCI to the culprit lesion of AMI (*n* = 154), (2) in-hospital death (*n* = 59), (3) without any follow-up data after discharge (*n* = 54), (4) second or more than second AMI during the study period (*n* = 68), (5) unable to take meals orally, (6) ≥2 vessels were treated simultaneously (*n* = 38), (7) unsuccessful PCI (*n* = 1). Our final study population was 1006 patients and was divided into the good appetite group (*n* = 860) and the poor appetite group (*n* = 146). The study flowchart is shown in Figure 1.

We compared the patients’ clinical characteristics in Table 1. Age was significantly older in the poor appetite group than in the good appetite group. The proportion of males was higher in the good appetite group than in the poor appetite group. BMI, diastolic blood pressure, albumin, and hemoglobin levels were significantly lower in the poor appetite group than in the good appetite group, and heart rate was significantly higher in the poor appetite group. The prevalence of hemodialysis was significantly higher in the poor appetite group than in the good appetite group. Killip class and modified KATZ index were more advanced in the poor appetite group than in the good appetite group.

Student’s *t*-test for normally distributed continuous variables. Mann-Whitney U test for abnormally distributed continuous variables. Chi-square test for categorical variables. Abbreviations: PCI = percutaneous coronary intervention, CABG = coronary artery bypass grafting, MI = myocardial infarction, ACE = angiotensin-converting enzyme, ARB = angiotensin II receptor blocker, GFR = glomerular filtration rate, BNP = brain natriuretic peptide, LVEF = left ventricular ejection fraction, ER = emergency room.

We compared lesion and procedural characteristics in Table 2. All parameters were comparable between the two groups.

Student’s *t*-test for normally distributed continuous variables. Mann-Whitney U test for abnormally distributed continuous variables. Chi-square test for categorical variables. Abbreviations: V-A ECMO = veno-arterial extracorporeal membranous oxygenation.

**Figure 2:**
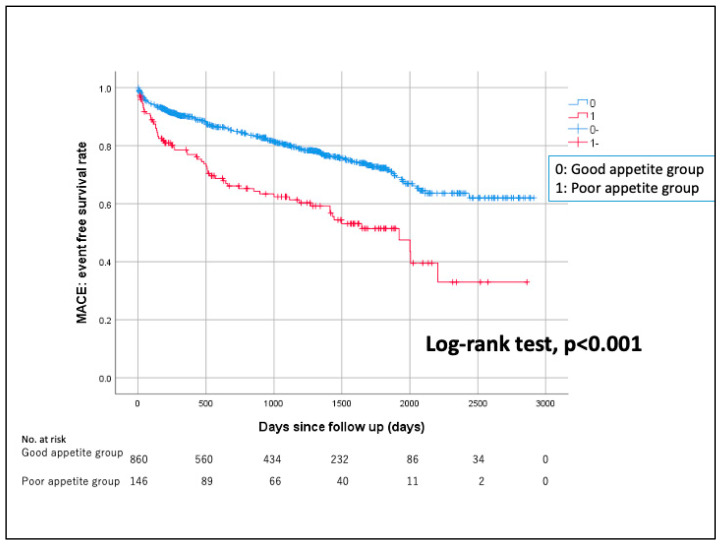
Kaplan–Meier curves for MACE between the two groups.

Kaplan–Meier curves for MACE between the two groups are shown in Figure 2. The median follow-up duration was 996 days (Q1: 262 days–Q3: 1588 days). The incidence of MACE was significantly higher in the poor appetite group than in the good appetite group.

We compared clinical outcomes between the two groups in Table 3, which also reveals the higher incidence of MACE in the poor appetite group.

The results of the multivariate Cox hazard analysis are shown in Table 4. MACE was significantly associated with poor appetite (HR 1.698, 95% CI 1.243–2.319, *p* < 0.001) after controlling for multiple confounding factors including age, sex, body mass index, diastolic blood pressure at admission, heart rate at admission, albumin, hemoglobin, hemodialysis, Killip class (1–3 vs. 4), and modified KATZ index (0–5 vs. 6).

In the adjusted model, poor appetite (vs. good appetite) was adjusted for age, sex, body mass index, diastolic blood pressure at admission, heart rate at admission, albumin, hemoglobin, hemodialysis, Killip class (1–3 vs. 4), and Modified KATZ index (0–5 vs. 6).

## 4. Discussion

The main findings of this study are as follows: (1) A total of 243 MACE were observed in the study population with a median follow-up duration of 996 days. (2) MACE was more frequently observed in the poor appetite group than in the good appetite group. (3) Multivariate Cox hazard analysis revealed that poor appetite was associated with MACE (HR 1.698, 95% CI 1.243–2.319, *p* < 0.001) after controlling for multiple confounding factors. Our results suggest that poor appetite might be a prognostic marker for long-term outcomes in patients with AMI.

Early studies showed that nutritional status is related to the long-term outcomes of heart failure or myocardial infarction [10,13]. Albumin is the most common indicator of nutritional status [24]. However, since serum albumin levels decrease in the presence of inflammation, the C-reactive protein (CRP)/albumin ratio (CAR) is proposed as an indicator of nutritional status in the acute phase of inflammatory diseases [25]. Moreover, CAR has been reported to be a predictor of long-term outcomes in patients with AMI [26]. The Geriatric Nutritional Risk Index (GNRI) and the Prognostic Nutritional Index (PNI) have been used for the indices of nutritional status, too [27,28]. Although these indices can evaluate nutritional status in a quantitative manner, these indices require blood test values such as albumin, CRP, lymphocyte count, and total cholesterol [9]. On the other hand, good or poor appetite is a simpler index that does not require blood test [15,16]. Furthermore, poor appetite precedes the deterioration of nutritional status [16,29]. Although there are some reports regarding the relationship between appetite and long-term outcomes in patients after TAVI or in patients with heart failure [15,16], there are few reports regarding that relationship in patients with AMI.

We should discuss why appetite was associated with the long-term outcomes of patients with AMI. Poor appetite might be associated with dehydration after discharge, which makes patients more susceptible to sudden cardiac death or non-fatal myocardial infarction [30]. In addition, patients with poor appetite might become undernourished [31], which can be a cause of various diseases including infection and heart failure [32,33]. There are possible several reasons for poor appetite in patients with AMI. One is that frailty was associated with poor appetite [34]. In our study, the modified KATZ index was higher in the poor appetite group than in the good appetite group, which indicates that frailty was more advanced in patients with poor appetite. The second reason is that poor appetite might reflect poor cardiac function. Cardiac dysfunction might cause intestinal edema and gastrointestinal tract dysfunction, which results in poor appetite [33]. The third reason is that poor appetite may be one of the manifestations of general poor psychological status and depression, which often accompany AMI [35]. Furthermore, depression may in turn influence poor outcomes of AMI. On the other hand, appetite is closely related to appetite hormones. Ghrelin, which is an orexigenic hormone primarily released by the stomach, is associated with perceptions of hunger in humans [36]. A small-sized randomized study showed that synthetic human acyl ghrelin improved cardiac output in patients with heart failure and reduced ejection fraction (HFrEF) [37]. Therefore, appetite hormones might play a role in the association between poor appetite and poor outcomes in patients with AMI.

The clinical implications of this study should be discussed. It is easier to evaluate appetite than to evaluate nutritional status. Not only physicians but also nurses can notice poor appetite. In general, it is recommended for patients with poor appetite to check gastrointestinal symptoms, taste disorders, and oral problems [38]. However, our results suggest that AMI patients with poor appetite should be followed up carefully as a high-risk group. Furthermore, if patients who had had good appetite before AMI had poor appetite, we should consider the possibility that poor appetite is caused by decreased cardiac function. The present study shed light on dietary intake on the day before discharge, which does not require any additional cost for evaluation, as a potential prognostic marker in patients with AMI. The strength of this study is to suggest a possibility of an unrecognized risk marker for predicting poor clinical outcomes in patients with AMI.

This study has the following limitations. First, this study was conducted retrospectively based on data from a single center. Thus, there are various potential selection biases. Because of the retrospective nature of this study, we might not adjust unknown confounding factors in multivariate analysis. Second, there were some variables with missing values. Third, dietary intake on the day before discharge might be influenced not only by appetite but also by patients’ preferences. It would be better to evaluate appetite by dedicated questionnaires [39]. However, since our study design was a retrospective study, it was not possible to evaluate appetite by using dedicated questionnaires after patients’ discharge. We used dietary intake on the day before discharge as a substitute for dedicated questionnaires. Fourth, we arbitrarily defined the cut-off value for good or poor appetite as 70% of dietary intake on the day before discharge, because there have been no clear cut-off values for poor appetite in patients with AMI.

## 5. Conclusions

Appetite at the time of discharge was significantly associated with long-term clinical outcomes in patients with AMI. Appetite can be evaluated non-invasively without additional cost. Patients with poor appetite should be carefully followed up after discharge from AMI. Future studies are warranted to confirm our findings in the setting of a multi-center registry or prospective cohort studies.

## Figures and Tables

**Table 1:** Patients’ characteristics between the good appetite group and the poor appetite group.

	All (*n* = 1006)	Good Appetite Group (*n* = 860)	Poor Appetite Group (*n* = 146)	*p*-Value
Age, years	69.5 ± 12.5	68.6 ± 12.6	74.5 ± 10.7	<0.001
male, *n* (%)	764 (75.9)	681 (79.2)	83 (56.8)	<0.001
Physical examination				
Body mass index (kg/m^2^)	24.3 ± 5.9	24.5 ± 6.2	23.1 ± 3.8	<0.001
Systolic blood pressure at admission (mmHg)	143.8 ± 31.6	144.6 ± 30.1	139.0 ± 36.0	0.056
Diastolic blood pressure at admission (mmHg)	82.5 ± 20.4	83.1 ± 19.8	78.8 ± 23.2	0.009
Heart rate at admission (beat per minute)	81.4 ± 22.2	80.5 ± 21.4	86.7 ± 25.9	0.001
Underlying disease				
Hypertension, *n* (%)	835 (83.0)	716 (83.3)	119 (81.5)	0.603
Diabetes mellitus, *n* (%)	547/1005 (44.1)	380/859 (44.2)	63 (43.2)	0.807
Dyslipidemia, *n* (%)	622/1005 (61.9)	531/859 (61.8)	91 (62.3)	0.906
Hemodialysis, *n* (%)	76 (7.6)	54 (6.3)	22 (15.1)	<0.001
History of previous PCI, *n* (%)	191 (19.0)	158 (18.4)	33 (22.6)	0.228
History of previous CABG, *n* (%)	34 (3.4)	27 (3.1)	7 (4.8)	0.306
History of previous MI, *n* (%)	119 (11.8)	103 (12.0)	16 (11.0)	0.725
Current smoker, *n* (%)	332/989 (33.6)	303/844 (35.9)	29/145 (20.0)	<0.001
Medication before admission				
Aspirin, *n* (%)	299/1002 (29.8)	252/856 (29.4)	47 (32.2)	0.502
Thienopyridine, *n* (%)	160/1002 (16.0)	135/856 (15.8)	25 (15.6)	0.680
Beta-blocker, *n* (%)	219/985 (22.2)	181/841 (21.5)	38/144 (26.4)	0.194
ACE-inhibitor, ARB, *n* (%)	378/986 (38.3)	316/842 (37.5)	62/144 (43.1)	0.208
Calcium channel blocker, *n* (%)	386/984 (39.2)	319/840 (38.0)	67/144 (46.5)	0.052
Statin, *n* (%)	349/990 (35.3)	292/846 (34.5)	57/144 (39.6)	0.239
Diuretic, *n* (%)	135/988 (13.7)	111/844 (13.2)	24/144 (16.7)	0.256
Hypoglycemic agents, *n* (%)	265/993 (26.7)	225/849 (26.5)	40/144 (27.8)	0.749
Insulin, *n* (%)	69/994 (6.9)	62/849 (7.3)	7/145 (4.8)	0.278
Laboratory data at admission				
Albumin (g/dL)	3.92 ± 0.52 (*n* = 1005)	3.96 ± 0.51 (*n* = 859)	3.69 ± 0.52	<0.001
Serum creatinine (mg/dL)	1.49 ± 2.07	1.39 ± 1.90	2.08 ± 2.84	<0.001
Estimated GFR (ml/min/1.73 m^2^)	63.5 ± 29.2 (*n* = 1005)	65.4 ± 28.8 (*n* = 859)	52.0 ± 29.4	<0.001
Hemoglobin (g/dL)	13.3 ± 2.13	13.5 ± 2.08	12.4 ± 2.16	<0.001
BNP (pg/mL)	385.4 ± 669.5 (*n* = 958)	334.8 ± 584.1 (*n* = 824)	696.2 ± 1000.4 (*n* = 134)	<0.001
LVEF, *n* (%)	53.1 ± 13.6 (*n* = 1002)	53.5 ± 13.3 (*n* = 856)	50.9 ± 15.0	0.070
Killip class				<0.001
1 or 2	832 (82.7)	731 (85.0)	101 (69.2)	
3	110 (10.9)	81 (9.4)	29 (19.9)	
4	64 (6.4)	48 (5.6)	16 (11.0)	
Cardiac arrest at prehospital or ER, *n* (%)	27 (2.7)	24 (2.8)	3 (2.1)	0.611
STEMI (vs NSTEMI)	576 (57.3)	503 (58.5)	73 (50.0)	0.055
Modified KATZ index				<0.001
0	506 (50.3)	471 (54.8)	35 (24.0)	
1	157 (15.6)	144 (16.7)	13 (8.9)	
2	124 (12.3)	101 (11.7)	23 (15.8)	
3	60 (6.0)	46 (5.3)	14 (9.6)	
4	43 (4.3)	31 (3.6)	12 (8.2)	
5	59 (5.9)	38 (4.4)	21 (14.4)	
6	57 (3.3)	29 (3.4)	28 (19.2)	

Student’s *t*-test for normally distributed continuous variables. Mann-Whitney U test for abnormally distributed continuous variables. Chi-square test for categorical variables. Abbreviations: PCI = percutaneous coronary intervention, CABG = coronary artery bypass grafting, MI = myocardial infarction, ACE = angiotensin-converting enzyme, ARB = angiotensin II receptor blocker, GFR = glomerular filtration rate, BNP = brain natriuretic peptide, LVEF = left ventricular ejection fraction, ER = emergency room.

**Table 2:** Lesion and procedural characteristics between the good appetite group and the poor appetite group.

	All (*n* = 1006)	Good Appetite Group (*n* = 860)	Poor Appetite Group (*n* = 146)	*p*-Value
Culprit lesion				0.501
Left main—left anterior descending artery, *n* (%)	498 (49.5)	422 (49.1)	76 (52.1)	
Right coronary artery, *n* (%)	345 (34.3)	294 (34.2)	51 (34.9)	
Left circumflex, *n* (%)	154 (15.3)	135 (15.7)	19 (13.0)	
Graft, *n* (%)	9 (0.9)	9 (1.0)	0 (0)	
Number of narrowed coronary arteries				0.308
1 vessel disease, *n* (%)	445 (44.2)	381 (44.3)	64 (43.8)	
2 vessel disease, *n* (%)	337 (33.5)	294 (34.2)	43 (29.5)	
3 vessel disease, *n* (%)	224 (22.3)	185 (21.5)	39 (26.7)	
Left main trunk lesion, *n* (%)	96 (9.5)	77 (9.0)	19 (13.0)	0.123
Initial TIMI flow grade of the culprit				0.768
0	373 (37.1)	322 (37.4)	51 (34.9)	
1	83 (8.3)	70 (8.1)	13 (8.9)	
2	163 (16.2)	142 (16.5)	21 (14.4)	
3	387 (38.5)	326 (37.9)	61 (41.8)	
Final TIMI flow grade of the culprit				0.934
0	0	0	0	
1	7 (0.7)	6 (0.7)	1 (0.7)	
2	17 (1.7)	14 (1.6)	3 (2.1)	
3	982 (97.6)	840 (97.7)	142 (97.3)	
Lesion length (mm)	15.4 ± 9.53	15.1 ± 9.04	16.7 ± 11.9	0.336
Reference diameter (mm)	2.54 ± 0.74	2.54 ± 0.75	2.51 ± 0.70	0.848
PCI procedure				0.191
Plain old balloon angioplasty, *n* (%)	37 (3.7)	29 (3.4)	8 (5.5)	
Aspiration only, *n* (%)	5 (0.5)	4 (0.5)	1 (0.7)	
Drug coating balloon angioplasty, *n* (%)	36 (3.6)	28 (3.3)	8 (5.5)	
Bare metal stent, *n* (%)	17 (1.7)	13 (1.5)	4 (2.7)	
Drug-eluting stent, *n* (%)	899 (89.4)	778 (90.5)	121 (82.9)	
POBA and aspiration, *n* (%)	6 (0.6)	4 (0.5)	2 (1.4)	
Other, *n* (%)	6 (0.6)	4 (0.5)	2 (1.4)	
Temporary pacemaker, *n* (%)	65 (6.5)	57 (6.6)	8 (5.5)	0.602
Intra-aortic balloon pumping support, *n* (%)	72 (7.2)	56 (6.5)	16 (11.0)	0.054
V-A ECMO, *n* (%)	10 (1.0)	9 (1.0)	1 (0.7)	0.684

Student’s *t*-test for normally distributed continuous variables. Mann-Whitney U test for abnormally distributed continuous variables. Chi-square test for categorical variables. Abbreviations: V-A ECMO = veno-arterial extracorporeal membranous oxygenation.

**Table 3:** Clinical Outcomes between the good appetite group and the poor appetite group.

	All (*n* = 1006)	Good Appetite Group (*n* = 860)	Poor Appetite Group (*n* = 146)	*p*-Value
MACE, *n* (%)	243 (24.2)	181 (21.0)	62 (42.5)	<0.001
All-cause death, *n* (%)	109 (10.8)	73 (8.5)	36 (24.7)	<0.001
Non-fatal myocardial infarction, *n* (%)	90 (8.9)	70 (8.1)	20 (13.7)	0.030
Re-admission for heart failure, *n* (%)	103 (10.2)	83 (9.7)	20 (13.7)	0.136

A chi-square test for categorical variables.

**Table 4:** Multivariate Cox hazard model to predict MACE.

Composite Endpoint	Hazard Ratios	95% Confidence Interval	*p* Value
MACE			
Good appetite	Reference		
Unadjusted poor appetite	2.204	1.651–2.941	<0.001
Adjusted poor appetite	1.698	1.243–2.319	<0.001
All-cause death			
Good appetite	Reference		
Unadjusted poor appetite	3.167	2.124–4.723	<0.001
Adjusted poor appetite	2.090	1.355–3.225	0.001
Non-fatal myocardial infarction			
Good appetite	Reference		
Unadjusted poor appetite	1.874	1.140–3.083	0.013
Adjusted poor appetite	2.023	1.187–3.448	0.010
Re-admission for heart failure			
Good appetite	Reference		
Unadjusted poor appetite	1.527	0.937–2.489	0.089
Adjusted poor appetite	1.124	0.669–1.889	0.658

## Data Availability

All data are available from the corresponding author on reasonable request.
